# xWAS analysis in neuropsychiatric disorders by integrating multi-molecular phenotype quantitative trait loci and GWAS summary data

**DOI:** 10.1186/s12967-024-05065-2

**Published:** 2024-04-25

**Authors:** Lingxue Luo, Tao Pang, Haohao Zheng, Chao Liufu, Suhua Chang

**Affiliations:** 1 Peking University Sixth Hospital, Peking University Institute of Mental Health, NHC Key Laboratory of Mental Health (Peking University), National Clinical Research Center for Mental Disorders (Peking University Sixth Hospital), 51 Huayuan Bei Road, Beijing, 100191 China; 2 Research Units of Diagnosis and Treatment of Mood Cognitive Disorder, Chinese Academy of Medical Sciences, Beijing, 100191 China

**Keywords:** Neuropsychiatric disorders, Quantitative trait loci, FUSION, SMR

## Abstract

**Background:**

Integrating quantitative trait loci (QTL) data related to molecular phenotypes with genome-wide association study (GWAS) data is an important post-GWAS strategic approach employed to identify disease-associated molecular features. Various types of molecular phenotypes have been investigated in neuropsychiatric disorders. However, these findings pertaining to distinct molecular features are often independent of each other, posing challenges for having an overview of the mapped genes.

**Methods:**

In this study, we comprehensively summarized published analyses focusing on four types of risk-related molecular features (gene expression, splicing transcriptome, protein abundance, and DNA methylation) across five common neuropsychiatric disorders. Subsequently, we conducted supplementary analyses with the latest GWAS dataset and corresponding deficient molecular phenotypes using Functional Summary-based Imputation (FUSION) and summary data-based Mendelian randomization (SMR). Based on the curated and supplemented results, novel reliable genes and their functions were explored.

**Results:**

Our findings revealed that eQTL exhibited superior ability in prioritizing risk genes compared to the other QTL, followed by sQTL. Approximately half of the genes associated with splicing transcriptome, protein abundance, and DNA methylation were successfully replicated by eQTL-associated genes across all five disorders. Furthermore, we identified 436 novel reliable genes, which enriched in pathways related with neurotransmitter transportation such as synaptic, dendrite, vesicles, axon along with correlations with other neuropsychiatric disorders. Finally, we identified ten multiple molecular involved regulation patterns (MMRP), which may provide valuable insights into understanding the contribution of molecular regulation network targeting these disease-associated genes.

**Conclusions:**

The analyses prioritized novel and reliable gene sets related with five molecular features based on published and supplementary results for five common neuropsychiatric disorders, which were missed in the original GWAS analysis. Besides, the involved MMRP behind these genes could be given priority for further investigation to elucidate the pathogenic molecular mechanisms underlying neuropsychiatric disorders in future studies.

**Supplementary Information:**

The online version contains supplementary material available at 10.1186/s12967-024-05065-2.

## Background

Neuropsychiatric disorders, including schizophrenia (SCZ), bipolar disorder (BP), major depressive disorder (MDD), attention deficit hyperactivity disorder (ADHD), and autism spectrum disorder (ASD) are highly heritable [1, 2], with numerous single nucleotide polymorphisms (SNPs) identified through genome-wide association studies (GWAS). However, the ability to interpret these variants has been hindered because many fall in non-coding regions of the genome or in regions of high linkage disequilibrium (LD) [3–5]. Given the non-coding characteristics of the majority of these variants, as well as their enrichment in known regulatory regions [6, 7], and conserved regions [8], many variants may function through the regulation of gene expression, splicing and even other modulation at the epigenetic level. As a result, it has motivated the development of methods to prioritize associated genes at GWAS loci by integrating multilevel molecular features.

One of the most prevalent methods is transcriptome-wide association studies (TWAS) [9], which integrates expression reference panels (eQTL datasets with expression and genotype) to discover gene-trait associations from GWAS datasets (with genotype) [10–12] whose responding expression data are lacking of. We can predict the gene expression of an individual based on the genetic profile from the GWAS cohort and estimate statistical associations [13] between ‘imputed’ gene expression and trait [14] by the correlation between expression and genotype of the eQTL cohorts in the individual-level GWAS data (such as PrediXcan [11]) or the summary-level GWAS data (such as Functional Summary-based Imputation (FUSION) [10], and S-prediXcan [12]). The methods represented by FUSION only identify the association of gene expression with trait, but summary data-based Mendelian randomization (SMR/HEIDI) [15], another summary-level tool, discovers the causal effect of gene expression on trait by conducting Mendelian randomization (MR) [15–17]. Both FUSION and SMR/HEIDI are popular and the most employed tools in TWAS analysis.

Besides, TWAS analysis has been extended from expression quantitative trait locus (eQTL) to other molecular phenotypes, such as splicing quantitative trait locus (sQTL) and protein quantitative trait locus (pQTL). Since effects of genetic variation on RNA splicing were demonstrated to contribute to complex disease risk in Li et al. [18], a well-powered sQTL analysis in developing human cortex with FUSION (called as splicing-wide association studies (SWAS)) and SMR/HEDI was first conducted by Walker et al. [19] to understand how functional genetic variates related with splicing impacts phenotypes. Wingo et al. is the first one to integrate depression GWAS results [20] with human brain proteomes [21] by performing a proteome-wide association study (PWAS) of depression, which integrated protein abundance reference pQTL datasets (with protein abundance and genotype) and discovered 20 novel proteins, which were not previously implicated in GWAS.

Many researchers also discovered other molecular-related risk loci based on epigenomic modulation, such as DNA methylation quantitative trait locus (mQTL) and N6-methyladenosine quantitative trait locus (m^6^AQTL). DNA methylation, an epigenetic marker, has been reported to play a critical role in many biological process and diseases [22–24]. Several methylation-wide association studies (called as MWAS) have been successful in identifying methylation loci associated with traits. Liu et al. performed a SMR/HEIDI test to explore putative pleiotropic methylation loci for Alzheimer’s disease (AD) neuropathology [25]. Different from DNA methylation, N6-methyladenosine (m6A), a most abundant modulation, happens at the mRNA level [26]. Dysregulation of m^6^A has been implicated in psychiatric disorders by previous studie s[27, 28]. For the first time, FUSION was used to report several risk m^6^A site in blood tissue associated with several neuropsychiatric such as SCZ, BP, and MDD (called as mRNA methylation-wide association studies, m^6^A-WAS). Their results revealed insights into mRNA m^6^A regulation, highlighting the important mechanism of m^6^A regulation in finding the m^6^A modulation-specific loci in GWAS [29].

With the increasing types of molecular phenotypes applied into the x-wide association-like study (xWAS) analysis, many genes corresponding to these molecular features had been obtained, but these results are independent of each other, making it difficult to have a general overview on these risk genes. As a consequence, a comprehensive summary is urgent to sort out these result genes corresponding to molecular features. In this study, we firstly summarized the information of kinds of xWAS studies including TWAS, SWAS, PWAS, MWAS about five neuropsychiatric disorders, involving SCZ, BP, MDD, ADHD, and ASD. Next, since xWAS analyses of some disorders have not been conducted with the latest GWAS dataset or absent for certain molecular phenotypes analysis, we performed a series of supplementary analyses to make the risk gene sets more complete. Based on the curated and supplemented results, we defined novel reliable gene lists and genes related with at least two types of risk molecular features of the five disorders. Then, we explored the functions of the novel genes by pathway enrichment. The genes mapped by more than one type of molecular were further explored for each order by defining multiple molecular involved regulation patterns (MMRP), which may promote understanding to pathogenic molecular mechanism underlying neuropsychiatric disorders.

## Methods

### Literature search strategy

**Fig. 1:**
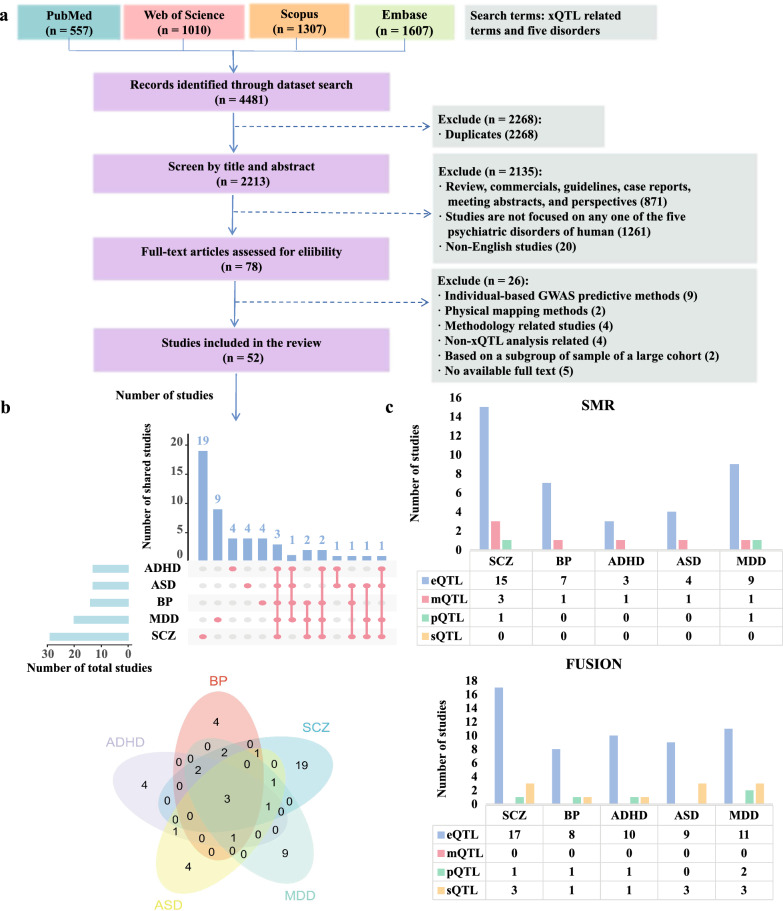
Overview of the included literature. **a** is an overview of the literature review process and statistics for the included studies; **b** is the studies for different disorders; **c** is the studies involving different types of QTL with two methods

A systematic search of the literature was performed in accordance to guidelines of the Preferred Reporting Items for Systematic Reviews and Meta-Analysis (PRISMA) statement [30]. In March 2023, four databases (PubMed, Web of Science, Embase, and Scopus) were searched for relevant articles from 1992 to 2023 with terms (SCZ, BP, ADHD, ASD, MDD related quantitative trait locus) by using (QTL OR “Quantitative Trait Loc*” OR “transcriptome wide association study” OR “proteome wide association study” OR “epigenome wide association study” OR TWAS OR PWAS OR EWAS) AND ("Schizophrenia"[Mesh] OR "Bipolar Disorder"[Mesh] OR "Depressive Disorder"[Mesh] OR "depression"[Mesh] OR "Attention Deficit Disorder with Hyperactivity"[Mesh] OR "Autism Spectrum Disorder"[Mesh]) in PubMed and without ‘[Mesh]’ in the terms in the other three databases. Our initial search identified 557 PubMed records, 1010 Web of Science records, 1607 Embase records, and 1307 Scopus records (Fig. 1). Duplicate records across these databases were identified to finally yield a total of 2213 independent records (Fig. 1).

### Literature selection criteria

Two authors (LXL, and HHZ) independently screened the titles and abstracts for the eligibility of studies using the Zotero software. Studies were excluded if they met the exclusion criteria: (1) review, commercials, guidelines, case reports, meeting abstracts, and perspectives, (2) studies not focused on any one of the five psychiatric disorders of human, (3) non-English studies. After applying these criteria, we retained 78 records (3.5%, 78/2213) and excluded 2135 records. For all 78 records, we reviewed the full-text articles to apply six additional exclusion criteria: (1) individual-based GWAS predictive methods (only summary-based GWAS predictive methods including SMR and FUSION-like methods were included; the latter includes FUSION, S-PrediXcan [12], Epixcan [31], MetaXcan [32], JTI [33], Mendelian randomization & joint-tissue imputation (MR-JTI) [33], UTMOST [34]), (2) physical mapping methods such as FOCUS [35], (3) methodology related studies, (4) non-xQTL related studies, (5) based on a subgroup of samples of a large cohort, (6) no available data due to inaccessible full text. Throughout the second filtering step, each article was screened independently by at least two of the authors (LXL, HHZ, CSH), and discrepancies were reviewed by an additional author, a consensus decision was taken by all the three authors. A total of 52 articles (66.7%, 52/78) were included for the xWAS analysis.

### Data extraction and collection

The following information were independently extracted from each eligible paper by two authors (LXL, and HHZ) who subsequently cross-checked the data. Discrepancies were resolved by discussion until a consensus was reached. The information included journal, xQTL type, xQTL dataset, tissue of xQTL, dataset of GWAS, analysis tools, adjustment method, threshold, number of total genes, and the names of genes mapped by the significant molecular features. For the genes obtained from the paper, we unified them into formatted gene symbols using the HUGO Gene Nomenclature Committee (HGNC, http://www.genenames.org/).

After extracting the results, we conducted comprehensive statistics on the studies for GWAS datasets, xQTL type, xQTL source. For each paper, we counted the times of both QTL datasets and GWAS dataset. For example, in a study with two QTL datasets, a mQTL dataset of Brain-mMeta and a pQTL dataset of ROSMAP were used to analyze the GWAS dataset of SCZ_2022, we counted it as one time for Brain-mMeta, ROSMAP, and SCZ_2022 separately. The detailed statistics results for GWAS datasets, weight files for FUSION, and xQTL datasets for SMR are presented in Additional file 1: Tables S1–3.

### Supplementary analyses

After sorting out the published studies, we found that for some diseases, some xQTL datasets were not analyzed for the latest GWAS dataset or absent for certain molecular phenotypes, which may hinder having a relatively comprehensive learning to the current results. Hence, we conducted supplementary analyses by using the latest GWAS summary dataset and the xQTL dataset with relatively bigger sample size (marked in Additional file 1: Tables S1–3). We selected the most two prevalent methods, FUSION and SMR, according to our curated results to get the reliable gene list and novel gene list for supplementary analyses results.

#### FUSION

We performed TWAS using GWAS summary statistics from the latest published SCZ [36], BP [37], ADHD [38], ASD [39], and MDD [20]. GWAS summary statistics were prepared for use in FUSION using the munge_sumstats.py script in LD Score Regression (https://github.com/bulik/ldsc). Given its localized pattern of long-range and complex LD, we excluded variants within the extended MHC region (chr6:28477797-33448354) to avoid spurious associations driven by the linkage disequilibrium pattern in this region. We combined reference weights with summary-level GWAS results to calculate the association between molecular phenotype and disease. The reference weights included expression panels (PsychENCODE [40] and Genotype Tissue Expression (GTEx V8_EUR) [41] downloaded from http://gusevlab.org/projects/FUSION/), splicing expression panel (CommonMind Consortium (CMC, http://gusevlab.org/projects/fusion/) [42]), protein abundance panels (Religious Order Study and Rush Memory and Aging Project (ROSMAP) [43], and Banner Sun Health Research Institute (Banner) [43]). Additionally, we utilized the m^6^A data and genotype data in the previous study [44] to compute weight following the description provided by FUSION. The Bonferroni-corrected *P* < 0.05 was used to correct for multiple comparisons.

#### SMR/HEDI

We supplemented xQTL related analyses for five disorders using SMR in which genetic variants were used as instrumental variables to evaluate the effects of molecular phenotypes on the variations of diseases. SMR analysis was carried out using the default parameters recommended by the developers. In this study, eQTL datasets were from PsychENCODE ( https://cnsgenomics.com/software/smr/#DataResource) [40] and eQTLGen (https://www.eqtlgen.org/cis-eqtls.html), sQTL from BrainMeta v2 (https://cnsgenomics.com/software/smr/#DataResource) [45] and GTEx V8_EUR (https://cnsgenomics.com/software/smr/#DataResource) [41], pQTL from ROSMAP (https://www.synapse.org/#!Synapse:syn23191787/wiki/606404) [46], mQTL from Brain-mMeta (https://cnsgenomics.com/software/smr/#DataResource) [47] and LBC_BSGS (https://cnsgenomics.com/software/smr/#DataResource) [48, 49], and m^6^AQTL were recalculated using fastQTL with nominal pass based on the original m6A peak data from Xiong et al. [44] and genotype data from dbGaP. The significant associations were determined by a Bonferroni-adjusted significance level to account for multiple comparisons. In addition, HEIDI test was also performed to test the presence of heterogeneity in the SMR association statistics and only genes passed HEIDI test (PHEIDI > 0.05) were retained.

### Discovery of reliable genes

We subsequently counted the supported evidence for each reported gene in a dictionary way, ‘GWAS dataset—xQTL type—tissue—tool—gene’. For example, if there is a gene called *SNX19* appearing in results of ‘SCZ_2014—eQTL—brain—FUSION’ and ‘SCZ_2014—eQTL—brain—SMR’, we considered the number of supported evidences of *SNX19* as twice. That means, GWAS datasets (published in different year, mainly from the Psychiatric Genomics Consortium (PGC)), xQTL types (including eQTL, sQTL, pQTL, mQTL), tissues (here, we grouped the tissues into brain and non-brain), tools (summary-based methods including FUSION-like and SMR) will affect the results, and the supported evidence of a gene will be calculated if there is any change in the variables corresponding to the evidence line of the gene. We defined the reliable genes as replicated at least twice.

### Checking novelty in GWAS

To determine the novelty of the reliable genes identified from the xWAS analyses, we identified the lowest p-values for the SNPs within 1 Mb upstream and downstream of each reliable gene using the summary statistics from the original traits GWAS [50]. The gene was defined as novel if the lowest p-values of the SNPs > 5e−8 in the original GWAS.

### Functional analyses for the novel genes

We combined the novel genes from curated literature and supplementary analyses for each disorder, and we performed functional enrichment analyses using gProfiler [51] and Functional Mapping and Annotation of Genome-Wide Association Studies (FUMA) [52] for novel gene list of each disease. Then, the enriched gene sets were grouped into functional groups by ClueGO (v2.5.9), which creates first a binary gene-term matrix with the selected terms and their associated genes. Based on this matrix, a term-term similarity matrix is calculated using chance corrected kappa statistics to determine the association strength between the terms. Since the term-term matrix is of categorical origin, kappa statistic was found to be the most suitable method. Finally, the created network represents the terms as nodes which are linked based on a predefined kappa score level [53]. The network is automatically laid out using the Organic layout algorithm supported by Cytoscape (v3.8.2) [54].

## Results

### Overview of published xWAS studies

Figure 1 presents a flow chart of the extraction of published xWAS studies. A total of 52 literatures were identified, of which 29, 13, 12, 12, and 19 were for SCZ, BP, ADHD, ASD, and MDD respectively (Fig. 1b). Among them, there were 13 (25.00%, 13/52) paper analyzed at least two of the five traits, three paper [55–57] involved five traits. Most of the analyzed GWAS datasets were from Psychiatric Genomics Consortium (PGC). For five disorders except ASD, the most utilized GWAS datasets were not the latest published version, for example, the one published in 2018 (called SCZ_2018) [35] was the mostly analyzed for SCZ, while its latest version is SCZ_2022 [36] (Additional file 1: Table S1).

As shown in Fig. 1c, most of the studies were only eQTL-related. Taking SCZ for example, 15 (78.9%, 15/19) were involved in eQTL for SMR, and 17 (80.9%, 17/21) for FUSION. It is noteworthy there was no analysis about mQTL with FUSION, and no sQTL-related studies with SMR regardless of diseases. For the dataset source of eQTL, we found most were from PsychENCODE, CMC and GTEx V7[58] (52.0%, 13/25 for SCZ in FUSION; 41.7%, 10/24 for SCZ in SMR). For other molecular phenotypes, please see Additional file 1: Table S2 for FUSION, Additional file 1: Table S3 for SMR. Moreover, only a few articles were involved in multiple QTL (20.1%, 6/29 for SCZ) (Additional file 1: Table S4), their combination form was listed in Additional file 2: Fig. S1. The results were almost similar for other diseases (Additional file 1: Tables S5–8). Apparently, previous xWAS studies mainly analyzed brain-related reference panels irrespective of the diseases and QTL types, though there were discrepancies in brain regions (Dorsolateral Prefrontal Cortex (DLPFC) were analyzed most) (Additional file 1: Tables S2–3).

**Fig. 2:**
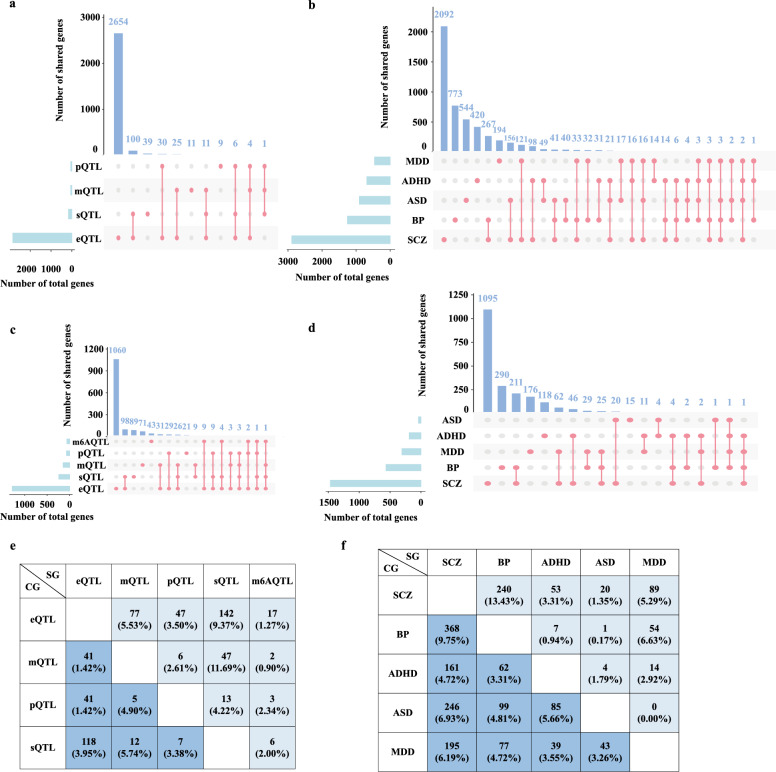
The number of results genes corresponding to five disorders from curated and supplemented analysis. **a**, **c** is the number of overlapping among different QTL of SCZ in curated genes (CG) and supplementary genes (SG) separately; **b**, **d** is the number of overlapping among different diseases in CG and SG separately; **e** is the contrast of overlapping ratio among different QTL of SCZ between CG and SG, left-lower part is for CG, right-upper part is for SG; **f** is contrast of overlapping ratio among different diseases between CG and SG, left-lower part is for CG, right-upper part is for SG

Then we sorted out the genes from curated analysis according to the method ‘GWAS dataset—QTL type—tissue—tool—gene’ of each disorder (we called these genes as curated genes (CG) in the following context) (Methods). Finally, there were 2890, 1253, 682, 904, 457 genes for SCZ, BP, ADHD, ASD, and MDD separately in total (Table 1). Taking SCZ for an example, most of the genes corresponding to splicing transcriptome, protein and DNA methylation could be well replicated by genes from eQTL, among which splicing transcriptome ranked top (Fig. 2a), other disorders were almost similar situation (Additional file 2: Fig. S2). Additionally, the validation among kinds of disorders varied (Fig. 2b), in which the replication ratio between SCZ and other disorders was much higher than the other pairs (Fig. 2f). It may originate from the much more result genes of SCZ, which increased the probability of overlapping with the other traits.

**Table 1:** Overview of curated genes (CG) and supplementary genes (SG). CG represents the genes from literature, SG represents the genes from supplementary analyses

Disease	Group	Total genes	Reliable genes (times > 1)	Novel reliable genes
SCZ	CG	2890	729	115
	SG	1510	560	42
	Total	3680	990	136
BP	CG	1253	126	62
	SG	579	169	46
	Total	1661	248	99
ADHD	CG	682	63	45
	SG	199	59	15
	Total	838	108	56
ASD	CG	904	48	41
	SG	40	12	7
	Total	924	50	41
MDD	CG	457	163	88
	SG	316	89	23
	Total	669	222	104

### Supplementary xWAS analyses

As we all known, the larger GWAS dataset is, the more risk loci will produce. However, according to our curated results, the latest GWAS dataset, which is also the largest one, has not yet been analyzed (Additional file 1: Table S1), which may discount the possibility to find more potential risk genes. Hence, we conducted supplementary analyses by using the latest GWAS dataset. In addition, we found the number of associated genes was nearly proportional with the sample size of the QTL panel, especially for the brain tissue in pre-analyses (Additional file 2: Fig. S3). As a result, we conducted analyses by FUSION and SMR with the reference molecular phenotype panels from brain and non-brain tissues according to its sample size and previous usage frequency (Methods, Table 2). On the other hand, the molecular phenotypes applied for xWAS varied for disorders, which made it less power to compare the replication ratio of QTL among diseases. Thus, we utilized the same QTL datasets for each disorder to validate whether the replication rate of molecular features among disorders was concordant. We calculated the total appearing times of a gene according to the method ‘GWAS dataset—QTL type—tissue—tool—gene’ of each disorder (Methods). The obtained genes from these supplementary analyses were called supplementary genes (SG) in the following context. Finally, there were 1510, 579, 199, 40, 316 genes for SCZ, BP, ADHD, ASD, and MDD separately in total (Table 1). Similar with CG, nearly half of the genes corresponding to other molecular features except m^6^A could be well replicated by genes from eQTL, with splicing transcriptome still ranked top (Fig. 2a for SCZ, Additional file 2: Fig. S2 for other disorders), but the replication ratio of SG were much higher than that in CG when we took denominator (the number of two types of molecular as denominator, and the number of their overlapping genes as numerator) into consideration (Fig. 2e). Additionally, the validation among kinds of disorders were also similar with that in CG (Fig. 2d). Different from the validation ratio among QTL, the replication ratio of SG was lower than that of CG (Fig. 2f), the conclusion held true in other diseases (Additional file 2: Fig. S5).

**Table 2:** Results of supplementary xWAS analyses for the five neuropsychiatric disorders conducted in this study

Method	xQTL Type	Datasets	Tissue	SCZ	BP	ADHD	ASD	MDD
FUSION	eQTL	GTEx_V8 (EUR)	Non-brain	5216	1566	237	197	592
			Brain	1396	398	68	111	157
		PsychENCODE	Brain	130*	41	2*	2*	26
	sQTL	CMC	Brain	149	45	4*	4	25
	pQTL	Banner	Brain	47	11	1	0	9
		ROSMAP	Brain	43	13	2	0	8
	m^6^AQTL	Brain	Brain	18	8	2	1	6
		Lung	Non-brain	25	7	0	0	2
		Muscle	Non-brain	18	6	1	0	4
SMR	eQTL	PsychENCODE	Brain	70*	24	3	3*	7
		eQTLGen	Non-brain	57*	22	3*	0*	18
	sQTL	BrainMeta v2	Brain	82	21	0	0	13
		GTEx_V8 (EUR)	Non-brain	1016	277	7	0	238
			Brain	288	60	10	0	57
	pQTL	ROSMAP	Brain	12	0	0	0	5
	mQTL	Brain-mMeta	Brain	139	32	0*	0	22
		LBC_BSGS	Non-brain	109	70	0	0	114
	m^6^AQTL	Brain	Brain	0	0	0	0	0
		Lung	Non-brain	1	0	0	0	0
		Muscle	Non-brain	0	0	0	0	0

The number denotes the number of significant genes; * denotes the results have been reported by published studies and the others were analyzed by this study

### Novel reliable genes in summarized gene list

Finally, we got 729, 126, 63, 48, 163 reliable genes (total supported evidence ≥ 2) in CG and 560, 169, 59, 12, 89 in SG for SCZ, BP, ADHD, ASD, and MDD, respectively. Then, we defined novel genes as those were not identified in original GWAS using the method of Wingo et al. [50] and the remaining of reliable genes were called non-novel genes. We obtained 136, 99, 56, 41, 104 novel genes for SCZ, BP, ADHD, ASD, and MDD separately after integrating genes from CG and SG (Table 2, Additional file 1: Tables S9–13). Among these reliable novel genes, there are 21, 26, 7, 12 genes were newly discovered genes by SG but not by CG for SCZ, BP, ADHD, MDD, respectively. Interestingly, the median value of replication times of novel genes was lower than that in non-novel genes both in CG and SG. This difference was significant in SCZ and BP (Additional file 2: Fig. S6). It implied that genes discovered at GWAS level were more frequently detected by various xWAS analyses.

### Functional exploration of novel reliable genes

**Fig. 3:**
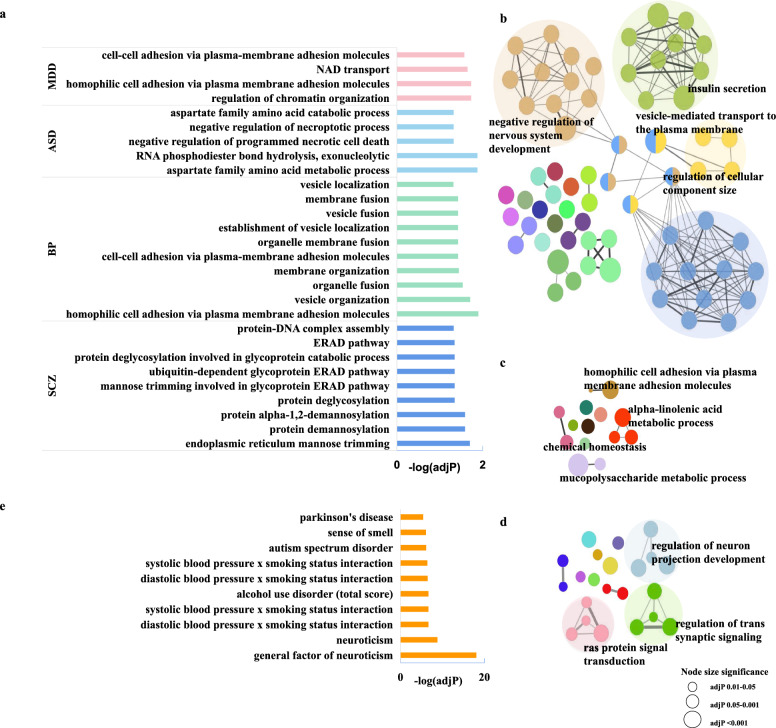
Pathways involved by the novel reliable genes from xWAS. **a** is GO-BP enrichment results with gProfiler for SCZ, BP, MDD and ASD. **b**–**d** provide insightful views of the interrelations between multiple GO-BP terms related with the novel genes of SCZ, BP and MDD, respectively. The network module in the same color represents the node terms are linked based on a predefined kappa score level. The size of the nodes reflects the enrichment significance of the terms. Functional groups represented by their most significant (leading) term are visualized in the network. **e** is the top ten of GWAS catalog terms associated with ASD

In order to explore the function of these novel reliable genes in xWAS analyses, we conducted gene-level functional mapping and annotation by gProfiler and focused on GO-BP term (with term size < 1000) [51] (Additional file 1: Table S14). The novel genes were significantly enriched in endoplasmic reticulum (ER)-associated protein degradation related pathways in SCZ. Many neurotransmitters transportation related pathways such as vesicles and membrane were enriched for novel genes of BP and MDD. In ASD, most of the novel genes were associated with aspartate family amino acid process. There was no GO-BP term found in ADHD (Fig. 3a).

We subsequently conducted functional enrichment with novel reliable genes by FUMA to explore more functions. However, we did not find significant enrichment for the five disorders. Thus, we further compared the enrichment results for the non-novel genes and all reliable genes (including both novel and non-novel genes) to explore probable functions of novel genes by filtering those enrichment functions only exist in all reliable gene sets but not in the non-novel gene sets. There were 103 GO-BP terms, 3 KEGG pathways and 106 GWAS Catalog terms (with term size < 500) (Additional file 1: Table S15) in total, which were considered as the possible biological functions caused by novel genes. The significant enrichment results of GO-BP terms for SCZ, BP, MDD were grouped into functional groups by ClueGO (v2.5.9) [53] (Fig. 3b–d). There was no significant GO-BP term result for ASD, but the novel genes were shown to be associated with other psychiatric diseases such as Parkinson disease (PD) in GWAS catalog (Fig. 3e). For ADHD, the novel genes were only enriched in GWAS catalog ‘body fat mass’. The correlations with other neuropsychiatric disorders for SCZ, BP, MDD were presented in Additional file 1: Table S15.

### Multiple molecular regulation pattern related risk genes

**Fig. 4:**
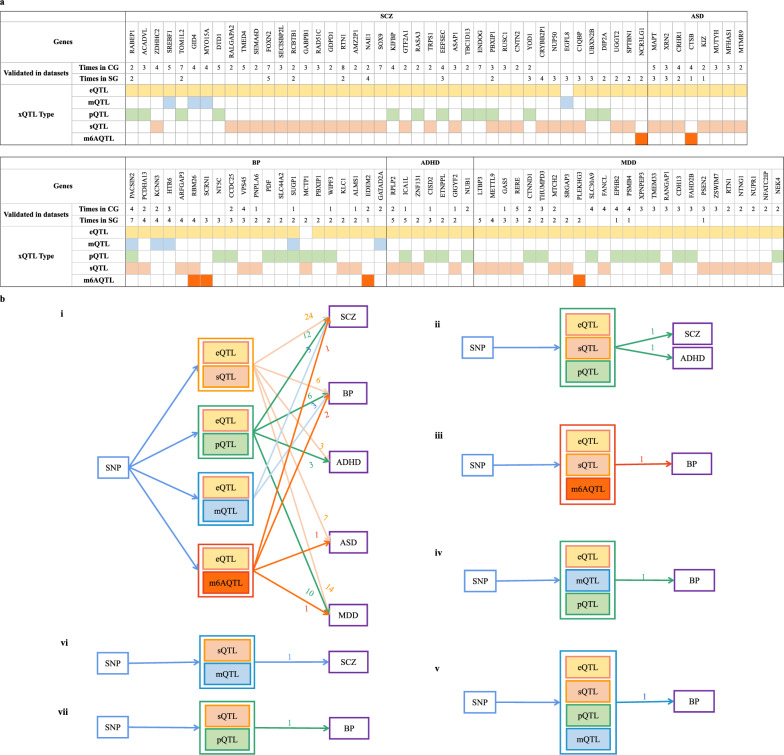
Multiple molecular regulation pattern related with the novel reliable genes. **a** Is an overview of the genes validated by at least two types of QTL from the xWAS analyses for the five psychiatric disorders. **b** Represents ten types of multiple molecular regulation pattern (MMRP) for the five psychiatric disorders. i–v show MMRP containing eQTL and vi–vii show MMRP without eQTL. Different colors represent corresponding molecular features. In order to have a clear view of the MMRP, the color of the line between QTL and disorders is concordant with the color corresponding to bottom QTL of each MMRP; and the number of genes involved in each MMRP are marked beside the lines

If there are more than one type of molecular phenotypes mapped by a gene through xWAS analysis, the gene may affect the disease through multiple regulation pattern. Hence, we defined these genes as multiple molecular regulation pattern (MMRP) related genes. We explored the regulation mode of genes based on the results of novel reliable gene lists (Fig. 4a). We totally obtained a summary of ten types of MMRP for the five disorders (Fig. 4b). We discovered most of the MMRP included only two molecular features (Fig. 4b.i, vi, vii), four types of MMRP involved more than two kinds of molecules (Fig. 4bii–v). Most of them were associated with eQTL, which may mean almost all the molecular effect that a gene suffers will ultimately relate to its gene expression, except Fig. 4bvi–vii. In addition, we observed BP was associated with the most types of MMRP, which also contained the only four-molecular regulation pattern (Fig. 4bv).

## Discussion

In this study, we summarized the xWAS studies of five neuropsychiatric disorders and the mapped genes corresponding to molecular phenotypes to have a comprehensive overview of these risk genes. Moreover, we supplemented series analyses, which enabled the risk gene list more complete. Finally, we integrated results from curated and supplemented analyses of five molecular phenotypes, elucidated the functions of the novel genes and identified ten types of MMRP, which may significantly contribute to unraveling the molecular regulatory mechanisms underlying this disease.

Regarding different molecular phenotypes, we observed that eQTL analysis obtained more risk genes compared to the other QTL, followed by sQTL both in CG and SG (Fig. 2a, c), which leads to phenomena that most of non-eQTL results could be replicated by eQTL, while less than half of the genes mapped to eQTL can be effectively replicated by alternative molecular features. Notably, the replication ratio of SG was higher than that in CG, indicating discrepancies in published xWAS studies or potential absence of certain molecular features for some disorders. One possible reason for this is that the larger sample size of the eQTL dataset leads to higher power for detecting eQTL compared to the other QTL. Nevertheless, the substantial replication across multiple molecular features may unveil intricate regulatory mechanisms that a gene might concurrently undergo.

Additionally, we compared the validation across disorders and observed contrasting phenomena where the replication ratio of CG was higher than that in SG (Fig. 2f), possibly due to the larger number of CG from some studies with unadjusted p-value threshold, which increased the likelihood of overlap. Interestingly, both in CG and SG, the median value of replication times was lower for novel genes compared to non-novel genes, with significant differences observed in SCZ and BP (Additional file 2: Fig. S6). To some extent, this reflects a higher probability for GWAS-significant genes to be influenced by molecular features while insignificant genes may be missed due to reduced statistical power at GWAS level but identified at xWAS level. Therefore, it is imperative to employ larger sample sizes xWAS analysis and consider multiple molecular phenotypes.

The novel genes associated with SCZ were significantly enriched in pathways related to endoplasmic reticulum (ER)-related protein degradation, an essential sub-cellular component involved in protein synthesis and post-translational modifications [59]. Previous studies have demonstrated the involvement of ER stress in the pathophysiology of SCZ, suggesting that targeting this pathway may potentially alleviate symptoms [60]. The pathways involved in the transportation of neurotransmitters, such as vesicles and membranes, were found to be enriched with novel genes associated with BP and MDD. These findings provide insights into potential mechanisms underlying the role of novel genes in psychiatric disorders. In ASD, a majority of the novel genes were linked to the metabolic process of aspartate family amino acids. Previous studies have demonstrated that antibodies against N-methyl-D-aspartate receptors (NMDAR) in the brain can lead to an autoimmune disease known as Anti-NMDAR encephalitis, which manifests with diverse psychiatric and neurological symptoms [61]. Therefore, targeting NMDAR may offer valuable insights into understanding the symptoms observed in MDD.

However, no significant results were observed in FUMA due to its stringent multiple testing corrections. Subsequently, we speculated on the potential functions of novel genes by filtering for enrichment functions present in reliable gene sets but absent in non-novel gene lists, which may indicate the contribution of these novel genes. The Gene Ontology (GO) biological process terms associated with SCZ were found to be related to negative regulation of nervous system development and insulin secretion. Notably, a previous study by Liu et al. [62] reported shared regulation of insulin secretion signaling between SCZ and type II diabetes (T2D), suggesting a possible comorbidity mechanism between these two disorders. Additionally, GO biological process terms were identified as being related to cell adhesion and chemical homeostasis, while KEGG pathway analysis revealed an enrichment in biosynthesis of unsaturated fatty acids including omega-3 polyunsaturated fatty acids (PUFAs). The use of n-3 PUFAs as mood stabilizers among bipolar disorder patients has been well validated in Rutkofsky et al. research findings [63]. Furthermore, axon guidance was found to be enriched in MDD, with neural functions previously implicated in the pathobiology of depression [64]. The results of Williams et al. demonstrated a significant decrease in the myelin Cross-sectional area (CSA) of splenium of the corpus callosum (spCC) axons in MDD [65] (Fig. 3b–d). These functional pathways can provide some hints for the pathogenic mechanism underlying these neuropsychiatric disorders.

Additionally, the ten types of MMRP found in this article are noteworthy. By integrating the results from multiple molecular features, we obtained a total of ten MMRP for the five disorders based on the novel and reliable gene lists. Most of MMRP consisted of only two molecular features (Fig. 4b.i) but there were several genes mapped by more than two molecular phenotypes, including *RBM26*, *PACSIN2*, *SUGP1* in BP, *PBXIP1* in SCZ, *ICA1L* in ADHD. We observed that BP exhibited the highest number of different types of MMRP among these disorders, consistent with its characterization as a dimensional phenotype [66]. We took *RBM26* in BP for discussion. *RBM26* is an RNA binding motif protein that participates in the polyadenylated RNA turnover in mammalian nuclei. The Poly(A) Tail eXosome Targeting (PAXT) connection promotes the recruiting process of the human ribonucleolytic RNA exosome to nuclear polyadenylated RNA. *RBM26*, as a new factor, is required for the PAXT function [67]. Though there is no direct evidence of *RBM26* and BP, two previous integrated analysis studies reported that *RBM26* acts as immune-related function not only in ASD [68], but also in non-psychiatric disorder including pancreatic cancer [69]. And there has been early researches showed that BP is accompanied by the dysregulation of immune-inflammatory pathways [70]. The gene is either found in the GWAS nor previous TWAS analysis, but show relations in our eQTL, m^6^AQTL and sQTL analysis which imply the effectiveness of application of multiple molecular phenotypes. Notably, *PACSIN2*, modulated by four distinct types of molecular phenotypes, is a member of the protein kinase C and casein kinase substrate in neurons family and its encoded protein plays a role in linking actin cytoskeleton with vesicle formation by regulating tubulin polymerization [71]. Although explicit reports regarding its functional involvement in BP remain elusive, it represents a promising candidate target gene deserving further investigation. Besides, from the remaining MMRP, we can get a hint that distinct molecular features may detect different risk genes. It is suggested multiple molecular features should be combined to help find more risk genes in the future.

It is important to acknowledge the limitations of this study. Firstly, it solely focused on five kinds of molecular phenotypes, while there exist numerous other molecular features that warrant our attention, which may modulate the variants through distinct mechanisms. Moreover, the regulation of many genes is related to spatial context, so QTL effects of a particular cell type in a given developmental stage might be shadowed in this analyses based on bulk tissue [19, 72]. Additionally, QTL data tend to be obtained from specific cohorts of individuals, it may influence the genes and gene functional enrichment results observed. For instance, the ROSMAP and Banner cohorts primarily consist of elderly individuals; therefore, when utilized as protein abundance panels for prediction in a cohort of young adults, we may observe an increased enrichment of genes associated with pathways characterized by Alzheimer's disease or Parkinson's disease.

## Conclusions

The analyses prioritized novel and reliable gene sets related with five molecular features based on published and supplementary results for five common neuropsychiatric disorders, which were missed in the original GWAS analysis. Besides, the involved MMRP behind these genes could be given priority for further investigation to elucidate the pathogenic molecular mechanisms underlying neuropsychiatric disorders in future studies.

### Supplementary Information

**Additional file 1: Table S1**. Basic information and used frequency of GWAS summary datasets of five diseases. **Table S2**. Basic information and used frequency of xQTL weight of five diseases for FUSION-like analyses. **Table S3**. Basic information and used frequency of xQTL sources of five diseases for SMR analyses. **Table S4**. Included literature information for SCZ. **Table S5**. Included literature information for BP. **Table S6**. Included literature information for ADHD. **Table S7**. Included literature information for ASD. **Table S8**. Included literature information for MDD. **Table S9**. Overview of novel genes in SCZ. **Table S10**. Overview of novel genes in BP. **Table S11**. Overview of novel genes in ADHD. **Table S12**. Overview of novel genes in ASD. **Table S13**. Overview of novel genes in MDD. **Table S14**. GO biological process (BP) functional enrichment results for the novel genes of five diseases with gProfiler. **Table S15**. Function enrichment of all reliable genes (novel reliable genes and non-novel reliable genes) of five diseases on GO-BP, GWAS Catalog, and KEGG using FUMA.

**Additional file 2: Figure S1**. Statistics for the studies involved one or multiple types of xQTL for the five diseases. **Figure S2**. Number of shared and specific genes among different xQTL in the curated genes (CG). **Figure S3**. The correlation of sample size of the reference panel with the number of significant genes for SCZ analysis using eQTL (A) and sQTL (B). **Figure S4**. Number of shared and specific genes among different xQTL in supplemented genes (SG). **Figure S5**. The contrast of overlapping among different xQTL of disorders between curated genes (CG) and supplementary genes (SG).**Figure S6**. Comparison between validated times of novel and non-novel genes respectively in curated genes (CG) and supplemented genes (SG).

## Data Availability

The GWAS datasets for xWAS analysis were downloaded from https://pgc.unc.edu/for-researchers/download-results/. The xQTL data were presented in the Method part of the manuscript. The codes for this article were stored in xQTL-analysis/code at main tuoyanghesuan/xQTL-analysis (github.com).

## Abbreviations

xWAS: X-wide association-like study
QTL: Quantitative trait loci
GWAS: Genome-wide association study
FUSION: Functional Summary-based Imputation
SMR: Summary data-based Mendelian randomization
MMRP: Multiple molecular regulation patterns
SCZ: Schizophrenia
BP: Bipolar disorder
MDD: Major depressive disorder
ADHD: Attention deficit hyperactivity disorder
ASD: Autism spectrum disorder
TWAS: Transcriptome-wide association studies
eQTL: Expression quantitative trait locus
sQTL: Splicing quantitative trait locus
pQTL: Protein quantitative trait locus
PWAS: Proteome-wide association study
mQTL: DNA methylation quantitative trait locus
m^6^AQTL: N6-methyladenosine methylation quantitative trait locus
MWAS: Methylation-wide association studies
AD: Alzheimer’s disease
MR-JTI: Mendelian randomization & joint-tissue imputation
GTEx: Genotype Tissue Expression
CMC: CommonMind Consortium
ROSMAP: Religious Order Study and Rush Memory and Aging Project
FUMA: Functional Mapping and Annotation
NMDAR: N-methyl-D-aspartate receptors
GO: Gene Ontology
T2D: Type II diabetes
PUFAs: Polyunsaturated fatty acids
