# Supplementary material for: xWAS analysis in neuropsychiatric disorders by integrating multi-molecular phenotype quantitative trait loci and GWAS summary data
Source: J Transl Med. 2024 Apr 25;22:387. doi: 10.1186/s12967-024-05065-2 (PMC11044291; doi:10.1186/s12967-024-05065-2)
Supplement: Supplementary file 2 — Additional file 2: Figure S1. Statistics for the studies involved one or multiple types of xQTL for the five diseases. Figure S2. Number of shared and specific genes among different xQTL in the curated genes (CG). Figure S3. The correlation of sample size of the reference panel with the number of significant genes for SCZ analysis using eQTL (A) and sQTL (B). Figure S4. Number of shared and specific genes among different xQTL in supplemented genes (SG). Figure S5. The contrast of overlapping among different xQTL of disorders between curated genes (CG) and supplementary genes (SG).Figure S6. Comparison between validated times of novel and non-novel genes respectively in curated genes (CG) and supplemented genes (SG). [file 12967_2024_5065_MOESM2_ESM.docx]

**Additional files**

Additional tables are in the excel file.

**Table S1**. Basic information and used frequency of GWAS summary datasets of five diseases.

**Table S2**. Basic information and used frequency of xQTL weight of five diseases for FUSION-like analyses.

**Table S3**. Basic information and used frequency of xQTL sources of five diseases for SMR analyses.

**Table S4**. Included literature information for SCZ.

**Table S5**. Included literature information for BP.

**Table S6**. Included literature information for ADHD.

**Table S7**. Included literature information for ASD.

**Table S8**. Included literature information for MDD.

**Table S9**. Overview of novel genes in SCZ.

**Table S10**. Overview of novel genes in BP.

**Table S11**. Overview of novel genes in ADHD.

**Table S12**. Overview of novel genes in ASD.

**Table S13**. Overview of novel genes in MDD.

**Table S14**. GO biological process (BP) functional enrichment results for the novel genes of five diseases with gProfiler.

**Table S15**. Function enrichment of all reliable genes (novel reliable genes and non-novel reliable genes) of five diseases on GO-BP, GWAS Catalog, and KEGG using FUMA. New terms mean the pathways were not included in the enrichment analysis by using only non-novel reliable genes.

**Figure S1**. Statistics for the studies involved one or multiple types of xQTL for the five diseases.


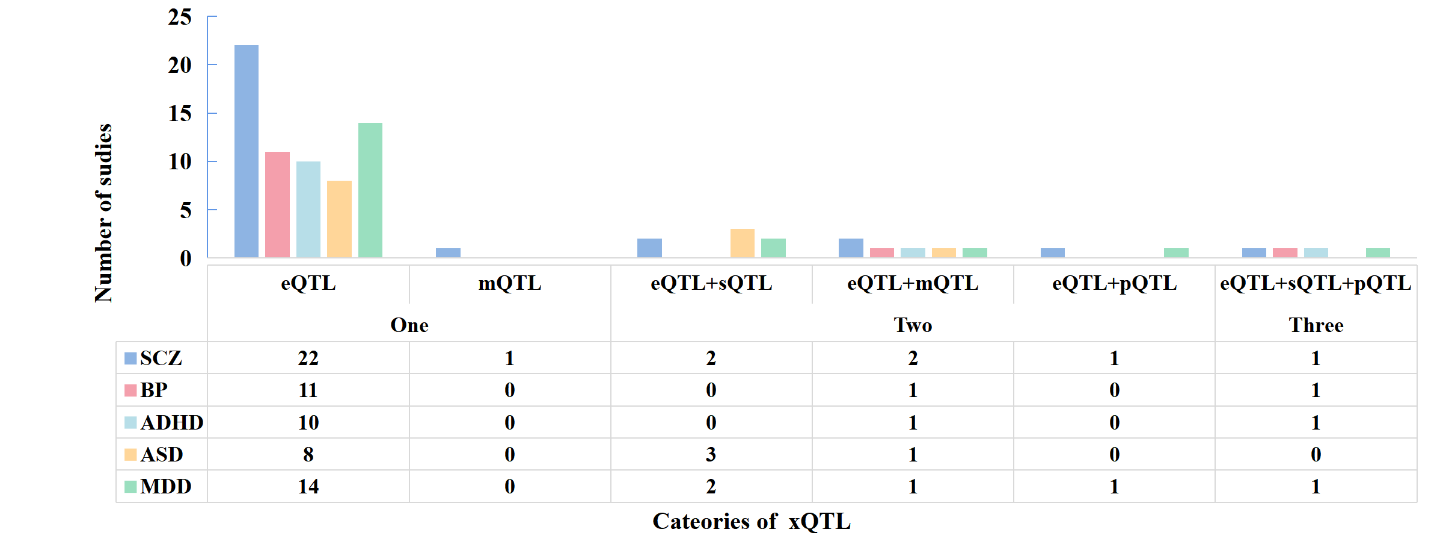


**Figure S2**. Number of shared and specific genes among different xQTL in the curated genes (CG). (A) for BP; (B) for ADHD; (C) for ASD and (D) for MDD.


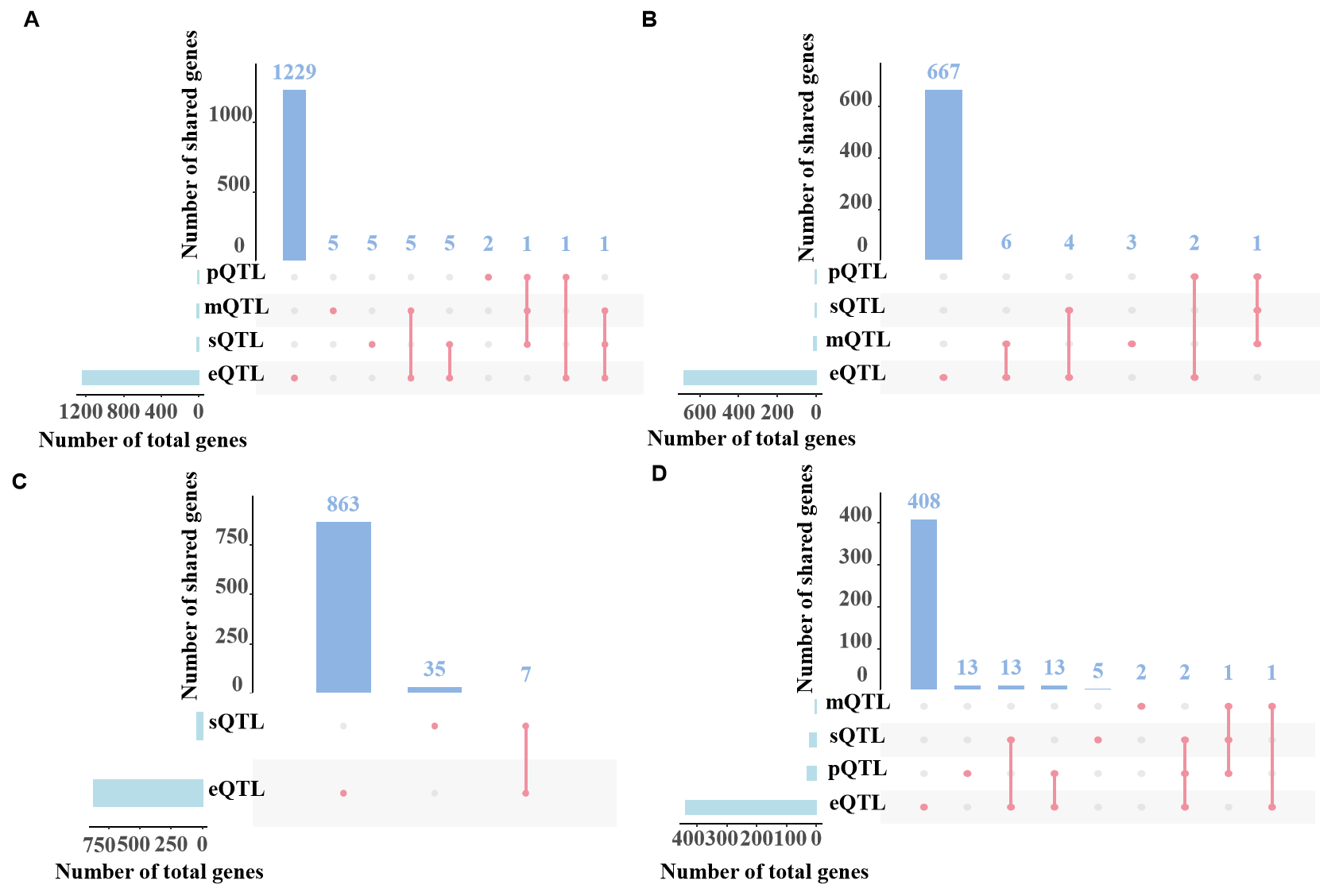


**Figure S3**. The correlation of sample size of the reference panel with the number of significant genes for SCZ analysis using eQTL (A) and sQTL (B). This analysis used SCZ_2022 GWAS dataset, eQTL used GTEx V8, sQTL used GTEx V8.

**Figure S4**. Number of shared and specific genes among different xQTL in supplemented genes (SG). (A) for BP; (B) for ADHD; (C) for MDD and (D) for ASD.

**
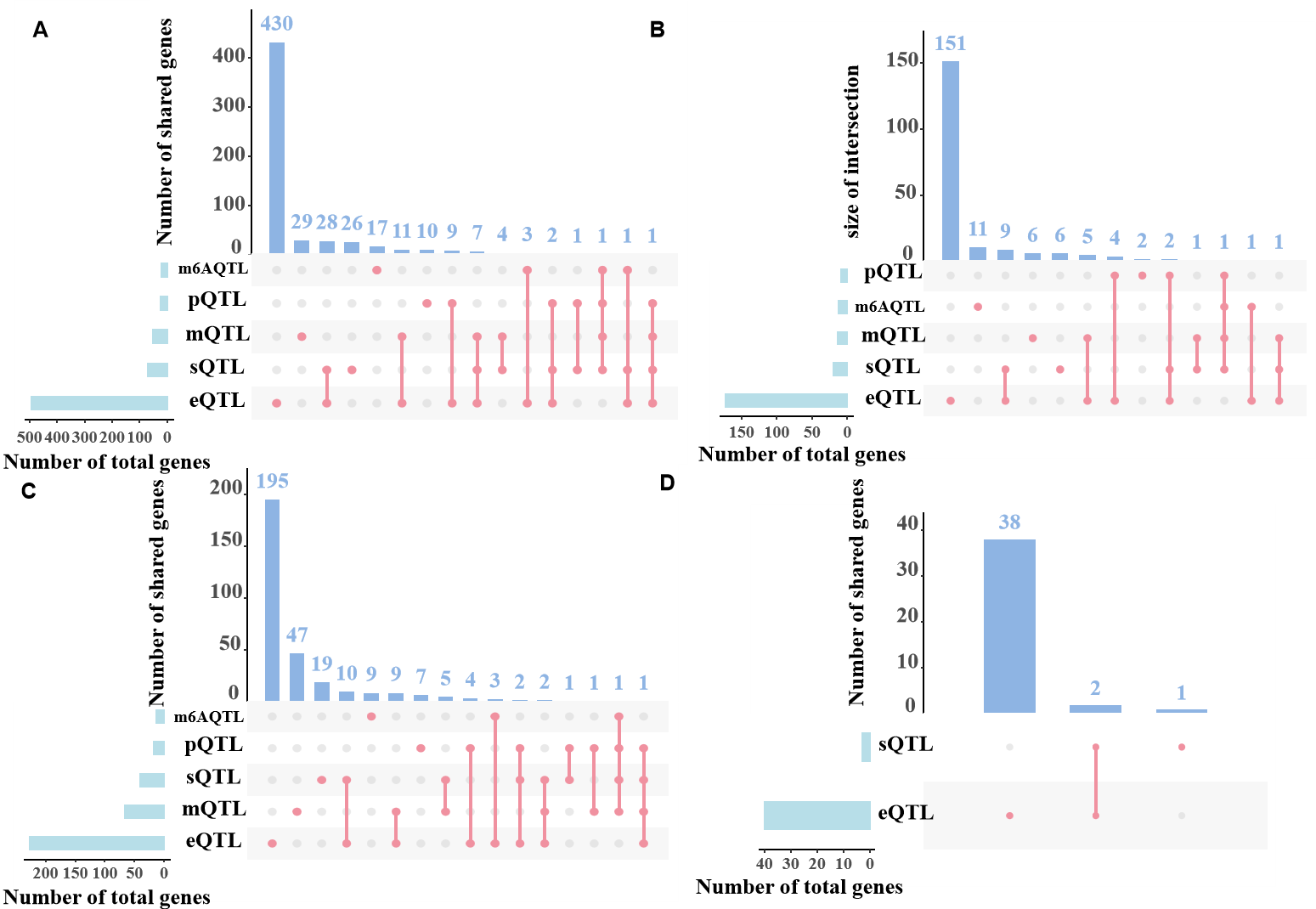
**

**Figure S5**. The comparison of overlapping among different xQTL of disorders between curated genes (CG) and supplementary genes (SG), left-lower part is for curated genes, right-upper part is for supplementary genes; (A) for BP; (B) for ADHD; (C) for MDD and (D) for ASD.

**
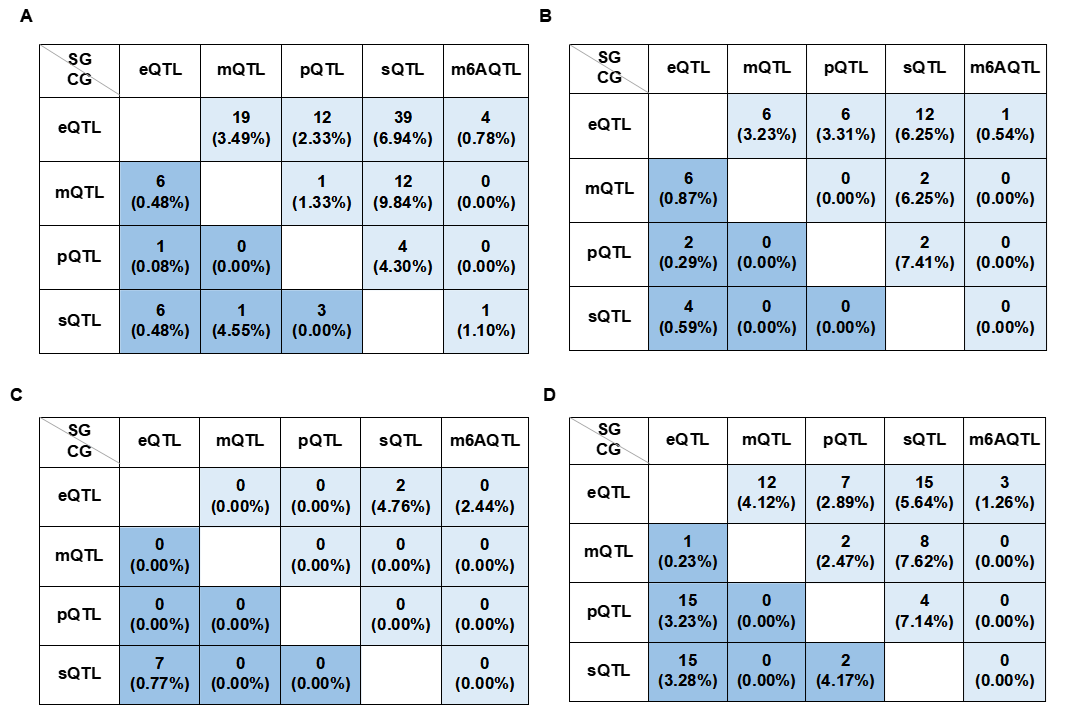
**

**Figure S6**. Comparison between validated times of novel and non-novel genes respectively in curated genes (CG) and supplemented genes (SG). (A) is the distribution of validated times of novel and non-novel genes respectively in CG and SG for SCZ. (B) is the comparison of median value of novel and non-novel genes respectively in CG and SG for SCZ. (C) is the distribution of validated times of novel and non-novel genes respectively in CG and SG for BP. (D) is the comparison of median value of novel and non-novel genes respectively in CG and SG for BP.
